# Regulation of the lysosome by sphingolipids: Potential role in aging

**DOI:** 10.1016/j.jbc.2022.102118

**Published:** 2022-06-09

**Authors:** Haiqing Tang, Xiaokun Huang, Shanshan Pang

**Affiliations:** School of Life Sciences, Chongqing University, Chongqing, China

**Keywords:** sphingolipid, lysosome, aging, life span, mTOR, lysosomal calcium, lysosomal cell death, lysosome–mitochondria communication, ALR, autophagic lysosomal reformation, Cer, ceramide, CerS, Cer synthase, ER, endoplasmic reticulum, GlcCer, glucosylceramide, GSL, glycosphingolipid, LCB, long-chain base, LCD, lysosomal cell death, LSD, lysosomal storage disorder, mTOR, mammalian target of rapamycin, NPC, Niemann–Pick disease type C, SM, sphingomyelin, Sph, sphingosine, S1P, sphingosine-1-phosphate, SPT, serine palmitoyltransferase, TOR, target of rapamycin, V-ATPase, vacuolar ATPase

## Abstract

Sphingolipids are a class of bioactive complex lipids that have been closely associated with aging and aging-related diseases. However, the mechanism through which sphingolipids control aging has long been a mystery. Emerging studies reveal that sphingolipids exert tight control over lysosomal homeostasis and function, as evidenced by sphingolipid-related diseases, including but not limited to lysosomal storage disorders. These diseases are defined by primary lysosomal defects and a few secondary defects such as mitochondrial dysfunction. Intriguingly, recent research indicates that the majority of these defects are also associated with aging, implying that sphingolipid-related diseases and aging may share common mechanisms. We propose that the lysosome is a pivotal hub for sphingolipid-mediated aging regulation. This review discusses the critical roles of sphingolipid metabolism in regulating various lysosomal functions, with an emphasis on how such regulation may contribute to aging and aging-related diseases.

Membrane lipids, primarily consisting of phospholipids, sphingolipids, and sterols, form a lipid bilayer that acts as a barrier between the cell and its environment and between different cellular organelles. Numerous recent studies indicate that these membrane lipids perform regulatory functions in addition to their structural roles. They participate in various cellular and organismal processes, such as signal transduction, organelle function, and animal physiology (1, 2, 3). Sphingolipids are a class of eukaryotic membrane lipids that are highly bioactive. Major subclasses of sphingolipids, including ceramide (Cer), sphingosine (Sph), and sphingosine-1-phosphate (S1P), are critical components of signaling pathways that regulate various cellular functions (4). In addition, major membrane sphingolipids such as sphingomyelin (SM) and glycosphingolipids (GSLs) are central components of specific membrane microdomains that serve as signaling platforms by recruiting specific membrane proteins. While it has long been recognized that many sphingolipid species play critical roles in cellular functions and organismal health, their mechanisms of action remain largely unknown.

Aging is a natural process that occurs in nearly all living organisms and is a significant risk factor for many chronic human diseases, including cancer, metabolic disorders, and neurodegenerative diseases. The aging process is accompanied by numerous metabolic changes, which has long been a focus of the aging community. For example, fatty acids, the central components of all eukaryotic lipids, undergo dramatic changes in several long-lived animals and humans, and numerous fatty acid species have been found to govern life span in model organisms (5). However, how these fatty acids regulate the aging process is largely unknown, as only a few of them have been found to act directly as signaling molecules in life span regulation (6). One of the essential functions of fatty acids is their incorporation into complex lipids as acyl chains. A recent study has identified a particular fatty acid that regulates life span by acting as the fatty acid chain of membrane GSLs (7). Accordingly, the levels of numerous sphingolipids vary in centenarians and long-lived animals, and the manipulation of sphingolipid metabolism can affect the life span of several model organisms (discussed in detail in a later section). While these studies suggest that sphingolipids play crucial roles in the aging process, the cellular mechanistic link between sphingolipids and aging is still a mystery.

Sphingolipid metabolism has great impacts on the functions of several organelles, including the lysosome. The lysosome is the cell’s primary degrading and recycling center, where damaged or nonessential organelles or molecules are degraded, and raw materials are released for resynthesis. In addition, the lysosome is recognized as a storage organelle for many metabolites and ions, such as amino acids, calcium, and iron, and thus plays crucial roles in maintaining nutrient homeostasis (8, 9, 10, 11). Many of these lysosomal functions are influenced by sphingolipids, which are particularly recognized in lysosomal storage disorders (LSDs), a group of human diseases characterized by the lysosomal accumulation of particular sphingolipid species, primarily GSLs (12). In LSDs, sphingolipid accumulation impairs the function of lysosomes, which in turn leads to a series of secondary cellular defects, such as protein aggregation and mitochondrial dysfunction (13). Moreover, increasing evidence indicates that sphingolipid-mediated regulation of lysosomes is not limited to LSDs but is observed in a variety of physiological or pathophysiological conditions associated with altered sphingolipid metabolism, including neurodegenerative diseases, cancer, immune regulation, and aging (4), indicating a broad and crucial role for sphingolipids in lysosomes.

Notably, the cellular defects observed in sphingolipid-related lysosomal disorders, such as autophagy stalling, protein aggregation, and mitochondrial dysfunction, are closely related to animal aging, implying that the mechanisms underlying these phenotypes may overlap. In addition, while the lysosome is considered an aging regulator because of its role in autophagy, recent research has linked a variety of lysosomal functions, such as lysosome regeneration, lysosomal degradation, and lysosomal cell death, to the regulation of aging and aging-related disease. And rising evidence indicates that these lysosomal processes are also regulated by sphingolipids. Thus, we propose that the lysosome is a critical hub through which sphingolipids act to control the aging process. In this review, we will summarize how sphingolipids regulate lysosomes, with an emphasis on lysosomal functions related to aging, and attempt to provide a perspective into the cellular mechanisms linking sphingolipids to aging. The current review will not discuss the direct effects of sphingolipids on other organelles, such as mitochondria.

## Sphingolipid metabolism and diversity

**Figure 1:**
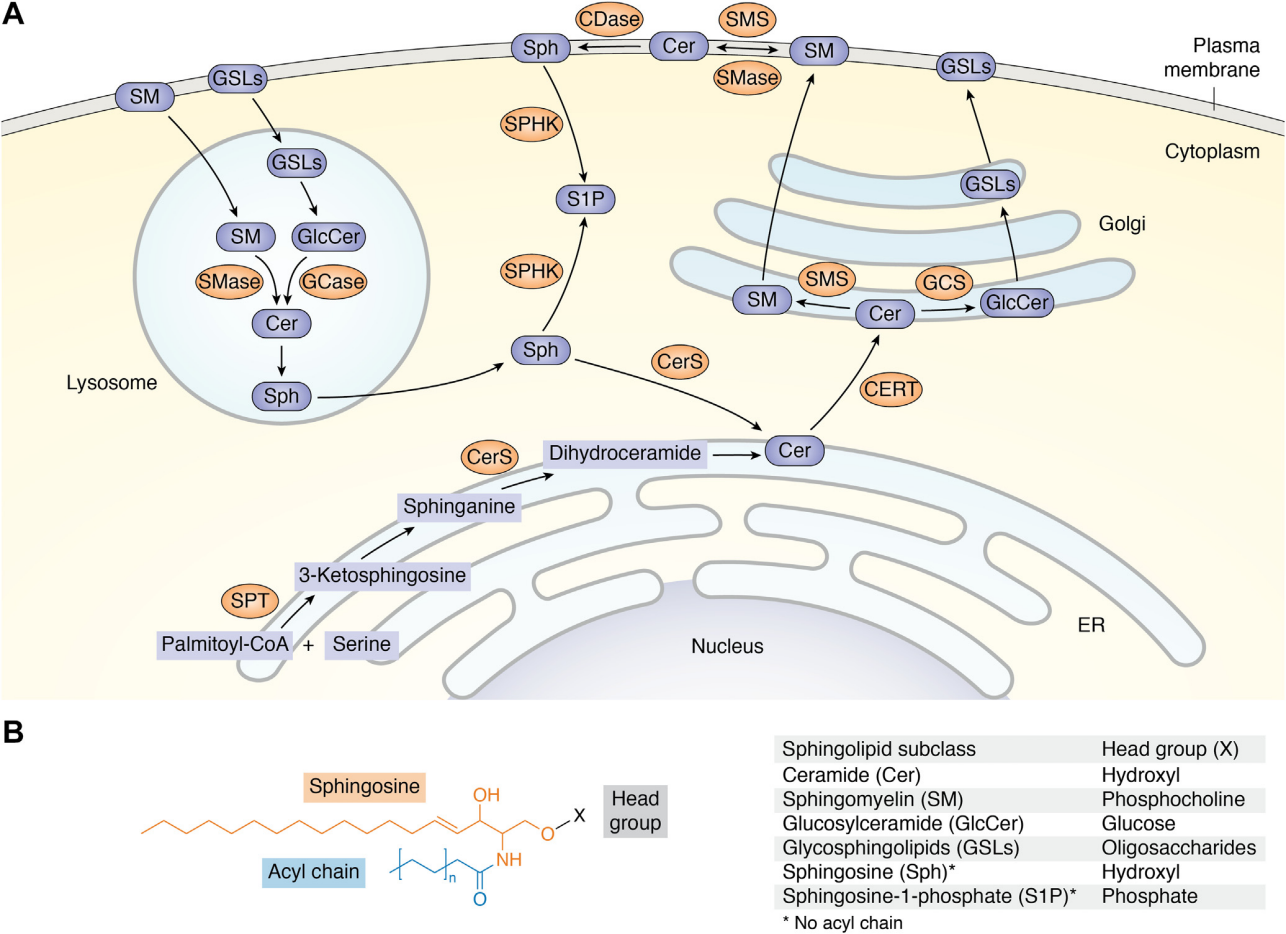
**Compartmentalization of sphingolipid metabolism.***A*, Cer is initially synthesized in the ER *via* the *de novo* synthesis pathway catalyzed by enzymes including SPT and CerS. After transport to the Golgi complex, it is further converted to SM and GlcCer by the enzymes SMS and GCS, respectively. GlcCer is then converted to other complex GSLs by adding additional carbohydrate groups. SM and GSLs are delivered to plasma membranes *via* vesicular transport. For breakdown, SM and GSLs are transported to lysosomes, where they are degraded to Cer by the actions of enzymes such as SMase and GCase. Cer is further degraded to Sph that is released to the cytoplasm for producing Cer and S1P. *B*, the structures of the sphingolipid subclasses. CDase, ceramidase; Cer, ceramide; CERT, ceramide transfer protein; CerS, ceramide synthase; ER, endoplasmic reticulum; GCase, glucosylceramidase; GCS, glucosylceramide synthase; GlcCer, glucosylceramide; GSL, glycosphingolipid; SM, sphingomyelin; SMS, sphingomyelin synthase; SMase, sphingomyelinase; S1P, sphingosine-1-phosphate; Sph, sphingosine; SPHK, sphingosine kinase; SPT, serine palmitoyltransferase.

Cer is the structural backbone of all sphingolipid subclasses. It is composed of a Sph long-chain base (LCB) and an N-acylated fatty acid chain with a carbon length of 14 to 26. Cer synthesis is initiated in the endoplasmic reticulum (ER) by serine palmitoyltransferase (SPT) that generates 3-ketosphinganine *via* the condensation of serine and an acyl-CoA, typically palmitoyl-CoA but may vary between organisms (14, 15). Following a further reduction of 3-ketosphinganine to sphinganine, Cer synthases (CerSs) attach a fatty acyl-CoA to form dihydroceramide, which is then desaturated to form Cer (Fig. 1*A*). It should be noted that most organisms have more than one CerS with distinct substrate specificity, resulting in diversity at the side-chain fatty acid level. This variety is critical for sphingolipid-dependent signaling, since numerous studies have demonstrated different roles of acyl chain–specific sphingolipids in a variety of biological processes, including aging (7, 16, 17, 18).

In addition, the diversity of sphingolipids can be attributed to the presence of different head groups. This occurs in the Golgi complex, where Cer is metabolized into other complex sphingolipid subclasses. Specifically, ceramidase removes the acyl chain from Cer to release Sph, which is phosphorylated at the head group to form the bioactive sphingolipid subclass S1P. Cer can also be converted to SM or GSLs by adding a phosphocholine head group or by adding various carbohydrate head groups (monosaccharides or oligosaccharides). These sphingolipids are then distributed to the plasma and organelle membranes *via* vesicular transport (Fig. 1, *A* and *B*).

The diversity of the head group is critical for maintaining sphingolipid homeostasis, as the functions and subcellular localizations of sphingolipid subclasses are generally distinct. To maintain such homeostasis, complex sphingolipids can be converted back to Cer *via* the salvage pathway. It begins with the transfer of complex sphingolipids such as SM and GSL to the late endosome/lysosome. Then they are degraded to Cer and Sph, which is released from lysosomes for regenerating S1P in the cytosol and Cer in the ER (Fig. 1*A*).

## Association between sphingolipids and aging

An imbalance between sphingolipid subclasses has been linked to several human disorders, including aging. Accumulating evidence indicates that the levels of numerous sphingolipid subclasses change with the aging process. In aged rats, Cer-generating enzymes exhibit higher activities than Cer-degrading enzymes, implying an accumulation of Cer (19). Interestingly, aged mice and *Caenorhabditis elegans* do not accumulate Cer but rather dihydroceramide (20, 21), the precursor of Cer (Fig. 1*A*). Consistently, the plasma levels of total dihydroceramide and major Cer species are lower in centenarians than in the aged group (mean age of 70 years) (22), suggesting that Cer accumulation may be a general indicator of aging.

Since aging results in an accumulation of Cer that may occur at the expense of SM by sphingomyelinase (Fig. 1*A*), aging may thus be associated with lower SM levels. Accordingly, increased SM levels may slow down the aging process. Consistent with this notion, long-lived animals or humans have higher SM levels. For example, two main SM species are significantly more abundant in long-lived naked mole rats than in laboratory mice (23). A serum profile study further indicates that the plasma of Italian centenarians contains higher concentrations of multiple SM species than that of the elderly (mean age of 76.4 years) (24), although the total SM levels appear to be unchanged in centenarians in Spain (22). As SM is a major component of plasma and organelle membranes, particularly membrane microdomains, the increased SM in long-lived organisms implies a critical function for SM-enriched membrane microdomains in healthy aging and longevity.

GSLs are another sphingolipid subclass that functions as critical components of specialized membrane microdomains such as lipid rafts (25). However, GSLs can accumulate in lysosomes when the lysosomal degradation of GSLs is impaired, as is the case with LSDs (12, 26). And in this regard, GSL accumulation is generally regarded as detrimental to organismal health. Indeed, GSL levels are increased in the brain and kidneys of elderly rodents (20, 27). Moreover, this increase can be prevented by caloric restriction, a conserved life span–extending paradigm (20). These findings imply that GSL accumulation may contribute to aging. However, conflicting findings were also reported. For instance, the levels of glucosylceramide (GlcCer), the simplest GSL and precursor to all other GSLs (Fig. 1*A*), are decreased in the immune cells of aged mice (28). In addition, major GSL species are higher in the plasma of centenarians than in the elderly (22). Since immune cells are abundant in plasma, it is conceivable that GSLs are necessary for proper immune function as we age. These findings suggest that, in certain circumstances, GSLs may be beneficial for longevity. The differences in GSL functions could be attributed to the distinct subcellular locations of GSLs. We propose that GSLs may accumulate in the lysosomes of brain cells and exert deleterious effects as observed in LSDs, which typically affect neuronal tissues. On the other hand, GSLs may be required as central components of membrane microdomains in certain cell types of aged organisms, such as immune cells.

## Life span regulation by sphingolipids

To better understand the roles of sphingolipids in aging, a series of studies examined how sphingolipid metabolism manipulations affect the life span of model organisms. In 1994, it was discovered that mutation of the yeast gene *lag1*, which encodes CerS, increased life span (29). Consistently, mutations of two sphingoid base kinase genes *lcb4* and *lcb5*, which lead to an increase in Cer precursor in yeast, reduce life span (30). In addition, downregulation of the yeast subunits of SPT, the rate-limiting enzyme in Cer synthesis, increases life span *via* a Sch9/S6K-dependent mechanism downstream of target of rapamycin 1 (TOR1)/mammalian target of rapamycin (mTOR) (31). These findings suggest that lowering Cer levels in yeast may promote longevity. However, it may not be the case in *Drosophila*, since mutations of the ceramidase genes increase Cer levels and promote organismal life span and stress resistance in flies (32). Also, the loss of function of Cer transfer protein, required for the maintenance of sphingolipid subclasses such as Cer, impairs stress resistance and decreases life span in *Drosophila* (33). Why do these studies conflict with each other? This may result from the distinct roles of different sphingolipid subclasses in life span regulation. Because Cer serves as the structural foundation for all other sphingolipid subclasses, manipulation of Cer may result in global changes in all sphingolipid subclasses, rendering the life span data inconsistent and difficult to explain. While Cer may be detrimental to longevity, other sphingolipid subclasses may act oppositely.

Studies in *C. elegans* support the aforementioned notion. Global inhibition of sphingolipid synthesis *via* mutation of the *sptl-1/spt* gene prolongs the life span of *C. elegans* (21). Loss of Sph kinase reduces the levels of S1P and significantly shortens life span (34), implying that the S1P subclass may act to promote worm longevity. Two additional studies investigated the roles of CerS in *C. elegans* life span regulation. Each CerS has unique substrate specificity and is thus responsible for the formation of Cer containing specific side-chain fatty acids. The genome of *C. elegans* encodes three CerS genes: *hyl-1*, *hyl-2*, and *lagr-1*. HYL-1 is required for the synthesis of sphingolipids with very long acyl chains (≥24), whereas HYL-2 produces sphingolipids with fatty acyl chains in the range of C20 to C22 (35). Mutation of *hyl-2* reduces life span (34, 36), whereas double mutations of *hyl-1* and *larg-1* increase life span (36). Consistent with the *hyl-2* life span result, our recent work found that sphingolipids containing the C22 fatty acyl chain are also required for longevity (7). In addition, we determined that C22 GlcCer is responsible for longevity and that it likely acts as a microdomain component on lysosomal membranes (7). These findings suggest that different sphingolipid subclasses, and even the same subclass with different fatty acyl chains, may play distinct roles in life span regulation.

## The lysosome is a critical hub for mediating the effects of sphingolipids on aging

Aging is associated with functional declines in numerous organelles, including the lysosome. The acidity, function, and homeostasis of lysosomes are all compromised as a result of aging, and preventing this decline extends the life span of model organisms (37, 38, 39). Accordingly, the enhancement of autophagy, which requires functional lysosomes to degrade ingested cargos, is recognized as a common mechanism in various life span–extension paradigms (reviewed in Ref. (40)). Moreover, mTOR, a key regulator of animal aging, is activated on lysosomal membranes and is thus influenced by lysosome homeostasis (41, 42, 43). In addition, lysosomal metabolites and ions, such as amino acids, calcium, and iron, play critical roles in the process of aging and aging-related diseases. For instance, when released from lysosomes, these molecules can affect the functions of secondary organelles, such as mitochondria, and thus have an indirect effect on aging *via* organelle communications (44, 45). Therefore, the lysosome is a crucial organelle for aging regulation.

**Figure 2:**
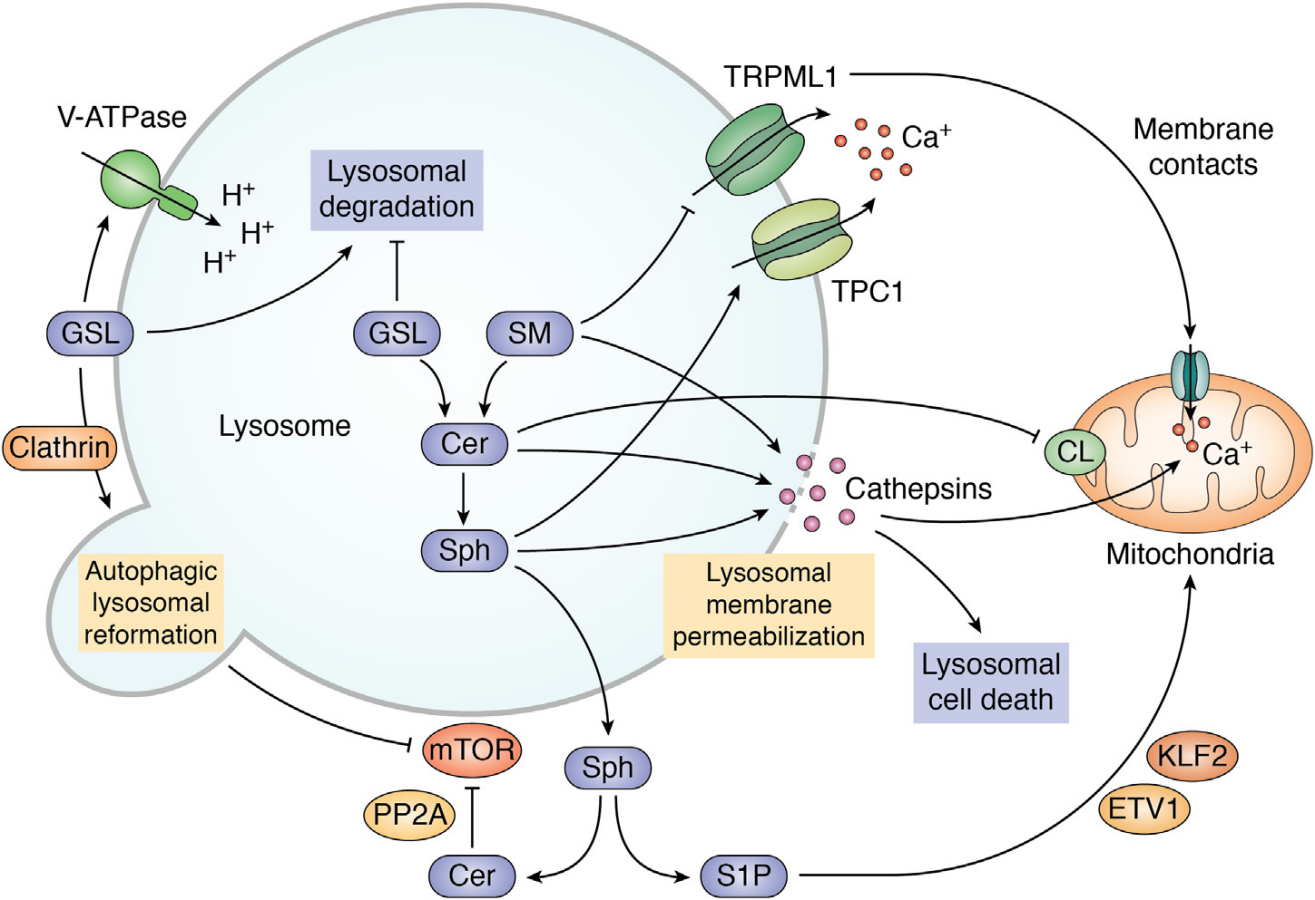
**Regulation of aging-related lysosomal functions by sphingolipids.** Lysosomal GSL accumulation impairs lysosomal degradation activity, whereas membrane-localized GSL is required for normal function of V-ATPase and thus for lysosomal activity; sphingolipids may be required for a low mTOR activity. Membrane-localized GSL suppresses mTOR *via* induction of autophagic lysosomal reformation (ALR), and Cer inhibits mTOR by activating PP2A; sphingolipid metabolites may inhibit lysosomal calcium release. Mechanistically, lysosomal SM can block the activity of the lysosomal calcium channel TRPML1, whereas Sph activates the calcium channel TPC1 and depletes lysosomal calcium storage; many sphingolipid metabolites, including SM, Cer, and Sph, can induce lysosomal membrane permeabilization (LMP) and subsequent release of cathepsins, which initiate lysosomal cell death; mitochondria are affected by lysosomes *via* multiple ways in response to fluctuations of sphingolipid levels. The lysosomal calcium TRPML1 and lysosome-released cathepsins can both regulate mitochondrial calcium levels. Lysosomal Cer suppresses mitochondrial function by decreasing cardiolipin (CL) levels. In addition, lysosome-derived S1P modulates mitochondrial biogenesis *via* a transcriptional program. Cer, ceramide; GSL, glycosphingolipid; mTOR, mammalian target of rapamycin; SM, sphingomyelin; S1P, sphingosine-1-phosphate; Sph, sphingosine; V-ATPase, vacuolar ATPase.

The lysosome is also essential for sphingolipid homeostasis. It is not only responsible for sphingolipid degradation (Fig. 1*A*) but also regulates their metabolism. For instance, mTOR regulates sphingolipid metabolism in yeast and mammalian cells by targeting the enzymes in sphingolipid synthesis (46, 47, 48, 49). The regulation between the lysosome and sphingolipids is mutual. Lysosomes can either respond to or be influenced by sphingolipid metabolites. Recent research indicates that sphingolipids regulate numerous aspects of lysosomes, not just in the context of LSDs. Thus, we will discuss the sphingolipid regulation of lysosomes in detail and propose how this might be integrated into aging regulation in the following sections. Several primary lysosomal functions/molecules are covered: lysosomal degradation, mTOR activity, lysosomal calcium, lysosomal cell death, and lysosomal-to-mitochondrial communication (Fig. 2).

## Regulation of lysosomal degradation by sphingolipid metabolism during aging

Lysosomal degradation activity is required for normal cell function and healthy aging. Lysosomal degradation acts as the last step of autophagy, whose activity declines with aging. Accordingly, the induction of autophagy has been well established as a universal longevity mechanism in model organisms (reviewed in Ref. (40)). Apart from autophagy, lysosomal function is also directly affected by aging. Lysosomal acidity, which is critical for its degradation activity, decreases during aging in yeast and worms (37, 39). Long-lived *C. elegans* reverse this decline *via* the transcription factor DAF-16/FOXO, which promotes lysosomal acidification by upregulating the expression of the vacuolar ATPase (V-ATPase) genes (37). In yeast, preventing this decline can also extend life span (39). Moreover, a recent study in *C. elegans* revealed that the properties of lysosomes, including morphology, dynamics, acidity, and degradation activity, undergo dramatic changes during aging but are well maintained in long-lived mutants. Moreover, impairing lysosomal function abolishes their longevity (38). In addition, lysosomal degradation activity is also activated by ER unfolded protein response and is required for its longevity effect (50). These studies indicate that lysosome activity is a crucial regulator of aging.

Regulation of lysosomal function by sphingolipids is well established in LSDs. In LSD patients with inherited deficiencies in GSL degradation enzymes or in assisted proteins, undegraded GSLs accumulate in lysosomes (12) and impair lysosomal homeostasis and degradation activity (13, 51). This lysosome-damaging effect of sphingolipid accumulation is not specific to GSLs. Cer accumulation in lysosomes also results in lysosomal expansion and stress in neuronal cells, which ultimately causes neuronal dysfunction (52). Then what about the process of aging? Notably, aging is associated with increased GSLs in the brain (20, 27), the primary organ affected by LSDs, implying that aging and LSDs may share certain mechanisms, particularly in neuronal tissues. Given the critical roles of lysosomes in neuronal function (53), GSL accumulation may impair lysosomal function in the aged brain, contributing to neuronal disorders associated with aging. A recent study reported that lysosomal activation can improve the function of quiescent neuronal stem cells during aging (54), supporting a crucial role for lysosomal function in brain aging. Further research into the relationship between GSL accumulation, lysosomes, and brain aging would be valuable and could shed new light on the aging process.

While it is believed that intralysosomal accumulation of GSLs impairs lysosomal function, several studies indicate that sphingolipid synthesis can be required for proper lysosomal function. The V-ATPase is a proton pump required for lysosomal acidity and thus for the activity of the lysosomal degradation enzymes. In yeast, disruption of sphingolipid synthesis dissociates a subdomain of the V-ATPase from vacuolar membranes, resulting in the dysfunction of the V-ATPase (55, 56). Similarly, mammalian melanocytic cells deficient in GSLs, possibly GlcCer, exhibit decreased V-ATPase activities and lysosomal acidity (57). These findings suggest that sphingolipid species on membranes, most likely GSLs, are required for the proper assembly or function of the lysosomal membrane proteins essential for normal lysosomal function. Our recent study in *C. elegans* substantiates this notion and elucidates its relevance to life span. Nematodes lacking GlcCer are short lived. The absence of GlcCer disrupts the membrane localization of clathrin, a protein essential for lysosome regeneration (58), causing aberrant giant lysosomes with impaired lysosomal degradation activity (7). Therefore, while intralysosomal GSL accumulation is detrimental, we propose that membrane-localized GSLs are required for lysosomal homeostasis and activity, which is critical for aging regulation.

## Sphingolipids regulate lysosomal mTOR signaling and its relevance to aging

mTOR is a negative regulator of aging. Its inhibition promotes longevity and improves healthy aging from unicellular eukaryotes to mice. As a nutrient sensor, mTOR activity is suppressed under conditions of nutrient scarcity and thus mediates the longevity effect of dietary restriction. mTOR controls the aging process *via* multiple cellular mechanisms, such as autophagy, translation, mitochondrial function, and cellular senescence (41, 42, 43).

mTOR activity is also influenced by sphingolipids, such as GSLs. Several studies suggest that GSL levels appear to be negatively correlated with mTOR activity. For instance, *Drosophila* and mammalian cells deficient in glucocerebrosidase (GBA1), the lysosomal enzyme responsible for the conversion of GlcCer to Cer (Fig. 1*A*), exhibit decreased mTOR activity, as indicated by a decrease in S6K phosphorylation (59, 60). In *C. elegans*, mutation of the glucosylceramide synthase gene (GCS), responsible for the generation of GlcCer from Cer, increases TOR activity and shortens life span (7). These findings suggest that GlcCer may act as a negative regulator of mTOR. However, opposite phenotypes were also reported, as *gba1* mutation is associated with hyperactivation of mTORC1 in induced pluripotent stem cell–neuronal cells (61), and GlcCer induces TOR signaling during development in *C. elegans* (62). This implies that GlcCer may regulate mTOR activity in a cell type– or developmental stage–dependent manner.

How does GlcCer regulate mTOR? It may involve autophagic lysosomal reformation (ALR), through which lysosomes are regenerated from autolysosomes. ALR is induced by mTOR, whereas inhibition of ALR results in feedback activation of mTOR (7, 63). In *C. elegans*, the absence of membrane-localized GlcCer impairs ALR and thus activates TOR (7), whereas a mammalian study reported that GlcCer accumulation inhibits mTOR and disrupts ALR and lysosomal homeostasis (60). The mutual regulation between mTOR and ALR may be critical for life span regulation in response to the fluctuations of GlcCer levels.

Apart from GSLs, other sphingolipid subclasses such as Cer also regulate life span through mTOR signaling. As mentioned previously, deficiency of SPT, the rate-limiting enzyme in Cer biosynthesis, prolongs yeast life span by inhibiting TOR activity (31). The SPT enzyme also regulates mTOR in cells from patients with hereditary sensory neuropathy type 1, a severe neurological disease caused by mutations in SPT encoding genes. In the hereditary sensory neuropathy type 1 rodent model, SPT deficiency causes mTOR activation by suppressing the phosphatase PP2A, a negative regulator of mTORC1 (64, 65). An intriguing question that merits further investigation is whether this Cer–PP2A–mTOR axis plays a critical role in aging and life span regulation.

## Sphingolipid regulation of lysosomal calcium: a possible role in aging

Calcium (Ca^2+^) is a critical signaling molecule that is involved in almost all aspects of cellular events and is involved in a variety of health and disease processes (66, 67), including aging (68, 69, 70). The ER is the primary intracellular compartment for calcium storage. The lysosome has also been discovered to have an intraorganellar calcium concentration comparable to the ER (71, 72). Lysosomal calcium signaling is required for various lysosomal functions, including autophagy and lysosome reformation (73, 74), two essential lysosomal events related to aging. Specifically, the release of lysosomal calcium activates calcineurin, which in turn binds and activates the transcription factor TFEB, a master regulator of autophagy (73). Lysosomal calcium, released by the channel TRPML1, controls lysosome tubulation and reformation *via* the calcium sensor ALG-2 and the ALG-2-associated dynactin–dynein motor (74).

Apart from these local effects, the interaction of the lysosome with other intracellular calcium stores enables the transduction of the lysosomal calcium event to other subcellular compartments and to a global calcium event (75, 76, 77), which activates the longevity transcription factor DAF-16 *via* the calcium-dependent kinases PKC-2 and SGK-1 (68, 69). Consistently, abnormal lysosomal calcium homeostasis is associated with aging-related diseases, such as Parkinson’s disease and Alzheimer’s disease (78, 79, 80). These studies indicate that lysosomal calcium may be a crucial regulator of aging.

The role of sphingolipids as calcium signaling regulators has been well established. Sphingolipids, especially S1P, can affect calcium homeostasis by modulating the plasma membrane calcium channels directly or by regulating the ER-located calcium channels indirectly (81). Nevertheless, the effects of sphingolipids on lysosomal calcium flux have only recently been discovered. Numerous sphingolipid subclasses, including Sph, GSLs, and SM, accumulate in lysosomes in LSD Niemann–Pick disease type C (NPC), accompanied by decreased lysosomal calcium storage and release (72). Reduced lysosomal calcium levels were also observed in LSD and Parkinson’s disease patients caused by a mutation in *gba1* (79). From a mechanistic standpoint, the decreased calcium release from lysosomes may result from the blockage of TRPML1, a primary lysosomal calcium channel, by SM accumulation in the lysosomes of NPC cells, although the level of lysosomal calcium remains unchanged in this study (82). A subsequent study established another mechanism for decreased lysosomal calcium storage and release: Sph accumulation induces calcium release by activating the calcium channel TPC1 in NPC cells (83). Notably, in a variety of LSDs, the disruption of calcium homeostasis is not only recognized in lysosomes but also in the ER (84, 85, 86). Such defects may result from lysosome-to-ER calcium signaling communication or, accumulated sphingolipids may escape from lysosomes and affect the ER-located calcium channels. Taken together, these findings strongly suggest that sphingolipid and lysosomal calcium signaling are inextricably linked. We argue that this type of interaction may play a critical role in aging regulation and warrants further investigation.

## Regulation of lysosomal cell death by sphingolipids and its relevance to aging

Lysosomal cell death (LCD) occurs as a result of lysosomal membrane permeabilization, which allows the release of lysosomal contents into the cytoplasm, including numerous proteases like cathepsins. Cathepsins can then cleave and activate the proapoptotic protein BID and initiate cell death (87). LCD has been implicated in aging and aging-related diseases. Several attributes of LCD, such as altered lysosomal membrane permeability and increased cathepsin levels, are observed in a variety of aging-related diseases, most notably neurodegenerative diseases (44), suggesting its potential role in brain aging.

Sphingolipids are an important class of molecules that regulate LCD. In LSD patients with GBA1 deficiency, lysosomal GSL accumulation is associated with increased cathepsin expression and their transport from lysosomes to the cytoplasm (88). Regulation of LCD by sphingolipids is not restricted to LSD-induced conditions. For instance, in mammalian cells, the absence of acid sphingomyelinase, the enzyme hydrolyzing SM to Cer, results in lysosomal membrane permeabilization, cathepsin B release, and final cell death (89). Also, Sph can act as a lysosomotropic detergent, destabilizing lysosome membranes and causing cell death (90). In addition, lysosomal overload of Cer could activate cathepsins B and D (91, 92), both of which can promote LCD (88). Therefore, lysosomal accumulation of certain sphingolipid subclasses induces LCD, which may contribute to aging-related disorders.

## Lysosome–mitochondria communication mediates sphingolipid effects on aging

Mitochondria play a critical role in the aging process. The decline in mitochondrial dynamics and function contributes to aging and aging-related disorders. Improvement of mitochondrial homeostasis or stress response could extend the life span of model organisms (93, 94). For instance, activation of the mitochondrial unfolded protein response, an adaptive program enabling mitochondrial proteostasis, extends life span in both worms and mice (95, 96).

Cer and other sphingolipid metabolites have been shown to act directly on mitochondria. Certain Cer species, such as CerS-generated C14/C16 Cer, impair mitochondrial dynamics and function by promoting mitochondrial fragmentation or mitophagy, resulting in a series of aging-related disorders (16, 18, 97). In addition, Cer can directly bind to the mitochondrial anion channel voltage-dependent anion channel 2 and induce mitochondrial apoptosis (98). While these studies demonstrate the direct effects of sphingolipid metabolites on mitochondria, recent research indicates that sphingolipids can also influence mitochondrial function indirectly *via* lysosome-to-mitochondria communications.

Defects in mitochondrial dynamics and functions have long been recognized in various LSDs (99, 100, 101, 102, 103), implying a possible connection between lysosomal sphingolipid accumulation and mitochondrial defects. For instance, in a mouse model of Gaucher disease, the most common LSD containing *gba1* mutation, mitochondria are severely fragmented and dysfunctional (103). It is widely accepted that abnormal mitochondrial function occurs as a passive result of impaired mitophagy in LSDs. Recent research suggests, however, that active communication between lysosomes and mitochondria may mediate the sphingolipid effects. Specifically, lysosomal proteins and metabolites, which are regulated by sphingolipids as discussed previously, can modulate mitochondrial homeostasis and function. For instance, mTOR is able to control mitochondrial dynamics, activity, and biogenesis by regulating the translation of essential mitochondrial proteins (104, 105). TRPML1, a lysosomal calcium channel that is inhibited by the sphingolipid SM (82), controls mitochondrial calcium uptake *via* lysosome–mitochondria contacts (77). In addition, Cer-activated cathepsin D was reported to induce a lysosome–ER–mitochondrial circuit that results in mitochondrial calcium overload and necrosis (106), suggesting LCD may act as a critical mediator between lysosomal sphingolipid and the ER/mitochondrial homeostasis.

Lysosome-derived sphingolipids can also affect mitochondrial function. In LSD patients, reduced lysosomal Sph release decreases cytoplasm S1P levels, which in turn suppresses mitochondrial biogenesis and function through the transcription factors KLF2 and ETV1 (107). Lysosomal sphingolipids can also regulate mitochondrial homeostasis *via* cardiolipin, a mitochondrial membrane phospholipid required for various aspects of mitochondrial functions (108, 109) and associated with aging (110, 111, 112, 113). NFYB-1, an inhibitor of lysosomal sphingolipid hydrolysis, was found to increase Cer in *C. elegans*, and this, in turn, impairs mitochondrial function and shortens life span by decreasing the levels of cardiolipin (114), which regulates life span in yeast and worms (113, 114). Thus, lysosomal sphingolipids may influence mitochondrial function *via* active signaling events, which is critical for life span determination.

## Conclusion and perspective

As reviewed here, the regulation of lysosomal molecules and functions by sphingolipids has advanced significantly in recent years (Fig. 2), and such regulation is critical in the development of aging and aging-related diseases. While these findings aid in our understanding of the cellular mechanisms linking sphingolipids to aging, they also raise a number of critical questions, including but not limited to the following: (1) What are the direct molecular targets of sphingolipid metabolites? For instance, can GSLs directly regulate the lysosomal V-ATPase or calcium channels? (2) There are still some inconsistencies regarding the actions of the same sphingolipid subclass. As discussed previously, this could be explained by the functional specificity conferred by the diversity of fatty acyl chain. Precise manipulation of specific sphingolipid metabolites may aid in resolving these paradoxes. This can also provide insights into a general biological question: why does the cell require so many complex lipids? (3) Do sphingolipids regulate any additional aging-associated lysosomal functions, including amino acids and iron homeostasis? (4) GSLs have a predominant effect on neuronal tissues in LSDs. Are neuronal GSLs critical for life span regulation?

The answers to these questions will provide a deeper understanding of how sphingolipids function. Although the lysosome is a critical hub for the sphingolipid regulation of aging, other organelles such as the ER and mitochondria are also regulated by sphingolipids. With the mutual communication between organelles, complex circuits and signaling networks must exist to mediate the effects of sphingolipids on aging. In this regard, elucidating the relationship between sphingolipids and lysosomes in the context of aging is critical but just serves as a starting point for this research field.

## Conflict of interest

The authors declare that they have no conflicts of interest with the contents of this article.
